# Obesity Is A Modifier of Autonomic Cardiac Responses to Fine Metal Particulates

**DOI:** 10.1289/ehp.9609

**Published:** 2007-02-26

**Authors:** Jiu-Chiuan Chen, Jennifer M. Cavallari, Peter H. Stone, David C. Christiani

**Affiliations:** 1 Department of Epidemiology, University of North Carolina School of Public Health, Chapel Hill, North Carolina, USA; 2 Department of Environmental Health, Harvard School of Public Health, Boston, Massachusetts, USA; 3 Cardiovascular Division, Brigham and Women’s Hospital, Boston, Massachusetts, USA; 4 Harvard Medical School, Boston, Massachusetts, USA

**Keywords:** air pollution, environmental health, heart rate variability, obesity, risk factors, susceptibility

## Abstract

**Background:**

Increasing evidence suggests that obesity may impart greater susceptibility to adverse effects of air pollution. Particulate matter, especially PM_2.5_ (particulate matter with aero-dynamic diameter ≤2.5 μm), is associated with increased cardiac events and reduction of heart rate variability (HRV).

**Objectives:**

Our goal was to investigate whether particle-mediated autonomic modulation is aggravated in obese individuals.

**Methods:**

We examined PM_2.5_-mediated acute effects on HRV and heart rate (HR) using 10 24-hr and 13 48-hr ambulatory electrocardiogram recordings collected from 18 boilermakers (39.5 ± 9.1 years of age) exposed to high levels of metal particulates. Average HR and 5-min HRV [SDNN: standard deviation of normal-to-normal intervals (NN); rMSSD: square-root of mean squared-differences of successive NN intervals; HF: high-frequency power 0.15–0.4 Hz] and personal PM_2.5_ exposures were continuously monitored. Subjects with body mass index ≥ 30 kg/m^2^ were classified as obese. Mixed-effect models were used for statistical analyses.

**Results:**

Half (50%) of the study subjects were obese. After adjustment for confounders, each 1-mg/m^3^ increase in 4-hr moving average PM_2.5_ was associated with HR increase of 5.9 bpm [95% confidence interval (CI), 4.2 to 7.7] and with 5-min HRV reduction by 6.5% (95% CI, 1.9 to 11.3%) for SDNN, 1.7% (95% CI, –4.9 to 8.4%) for rMSSD, and 8.8% (95% CI, –3.8 to 21.3%) for HF. Obese individuals had greater PM_2.5_-mediated HRV reductions (2- to 3-fold differences) than nonobese individuals, and had more PM_2.5_-mediated HR increases (9-bpm vs. 4-bpm increase in HR for each 1-mg/m^3^ increase in PM_2.5_; *p* < 0.001).

**Conclusions:**

Our study revealed greater autonomic cardiac responses to metal particulates in obese workers, supporting the hypothesis that obesity may impart greater susceptibility to acute cardiovascular effects of fine particles.

Altered autonomic cardiac activities, such as heart rate (HR) increases and overall heart rate variability (HRV) reduction (Zareba et al. 2001), in response to particulate matter (PM) exposure have been hypothesized as one of the major mechanistic pathways for PM-related adverse cardiac events. As reported in a recent extensive review on the health effects of fine particulate air pollution (Pope and Dockery 2006), PM exposure has generally been found to be associated with declines in most HRV measures, suggesting adverse effects on cardiac autonomic function. Pope and Dockery (2006) also noted that understanding of who is most at risk or susceptible is one of the most important gaps in our current knowledge regarding PM-related health effects.

The marked increases in prevalence of overweight and obesity over the last two decades in the United States has imposed a major public health concern (Hedley et al. 2004). Recent research findings point to the possibility that obesity may impart greater susceptibility to the adverse effects of PM exposure. In an inhalation study of healthy children 6–13 years of age, body mass index (BMI) was associated with a graded increase in the estimated total lung dose of deposited fine particles (i.e., deposited particles/time) (Bennett and Zeman 2004). In a panel study of 44 senior citizens, vascular inflammatory response (measured by C-reactive protein) to ambient levels of PM_2.5_ (particulate matter with aerodynamic diameter ≤2.5μm) averaged over 1–7 days is greater in obese (BMI ≥ 30 kg/m^2^) than in nonobese subjects (Dubowsky et al. 2006). However, no prior studies have examined the differential autonomic cardiac responses in obese versus nonobese individuals.

The objective of this study is to investigate whether autonomic cardiac responses to metal particulates are aggravated in the obese sub-population. We hypothesized that obese participants would experience greater autonomic modulation than those without obesity.

## Materials and Methods

### Study population

The study was approved by the Human Subject Committee of Harvard School of Public Health, and written informed consent was obtained from each participant. The study population came from a cohort of boilermakers in eastern Massachusetts. Between 2003 and 2004, subjects were recruited to participate in a study to assess acute cardiopulmonary and inflammatory responses to particulates. None met any predetermined exclusion criteria (unstable angina, bundle branch or atrioventricular block, atrial fibrillation or flutter, or other rhythms or clinical conditions compromising HRV analysis). Of 35 eligible workers who had completed physical examination (medical histories, weight and height measurement, and resting blood pressure), 20 volunteered for the intensive monitoring of autonomic cardiac activities, personal PM_2.5_ exposures, and daily activities for 24–48 hr during the same winter period (25 January to 8 February in 2003 and 31 January to 8 February in 2004). There were two defective ambulatory electrocardiogram (AECG) recordings, leaving 18 subjects included in the current study. All workers were at a welding school on the sampling day. This indoor work environment was well ventilated and temperature controlled, and workers were exposed to fine particulates while arc welding, grinding, cutting, or involved in other activities on mild steel. We have previously documented high levels of fine metal particulates in the studied work-place (Kim et al. 2003; Liu et al. 2005).

### Personal characteristics

We used a modi-fied American Thoracic Society questionnaire (Ferris 1978) to collect information on respiratory symptoms, personal medical histories, and current use of medication. Also, we solicited information on demographic features, lifestyle factors (e.g., smoking, drinking, exercise), and recent occupational activities. Self-reported status of diabetes mellitus and smoking was furthered verified by structured interviews and urine tests for sugar and cotinine. Because both HRV and PM exposure levels are potentially affected by other activities, all workers were asked to record the times when they performed different occupational activities in the workplace and also times spent in usual daily activities, such as cigarette smoking, coffee drinking, eating, alcohol consumption, exercising, and sleeping. All participants had their body weights (kilograms) and standing heights (meters) measured in the early morning by trained personnel. Subjects were classified as obese if their calculated BMI was ≥ 30 kg/m^2^. Two to three blood pressure determinations were made by the same physician after subjects had been sitting and resting for 10–15 min before the work shift, and the average was used for analyses.

### AECG monitoring

AECG recordings (24-hr or 48-hr) were performed using Applied Cardiac Systems AM cassette recorders (Laguna Beach, CA). Recorded signals from two leads (aVF and modified V_5_) were synchronized with personal air samplers. Recordings were analyzed in the AECG Core Laboratory at Brigham and Women’s Hospital.

### Measurement of autonomic cardiac activities

We used HRV and HR as measures of autonomic cardiac activities in response to PM exposure. Using a Marquette MARS Workstation (Milwaukee, WI), an AECG research specialist reviewed and, when necessary, corrected automatically determined categorization of QRS complexes into normal or ectopic beats. After regions of noise and artifact were eliminated, software facilities on the MARS were used to export beat timing and annotation information for analysis and creation of response variables through customized PC-based software written in C-language. Only normal-to-normal (NN) intervals between 150 and 5,000 msec with NN ratios between 0.8 and 1.2 were submitted to HRV analyses. Linear interpolation was constructed to replace missing beats including the removed ectopic beats and epochs with noise and artifacts. All HRV measures were computed on each 5-min epoch from a rate tachogram constructed from acceptable NN intervals (Berger et al. 1986). For time-domain parameters, including SDNN (standard deviation of NN intervals; in milliseconds), rMSSD (square root of the mean squared differences of successive NN intervals; in milliseconds) and average HR (in beats per minute), the tachogram gaps were set to the mean tacho-gram rate over all available intervals to avoid spurious variance that might result from interpolation, and all variance measures were appropriately scaled for the available tacho-gram duration. We used the high-frequency (HF) power of HRV (0.15–0.4 Hz) as the index of vagal activity. Our quality control data indicated an excellent agreement (intra-class correlation coefficient > 0.95) between results of repeated analyses for all time-domain and frequency-domain parameters.

### Measurement of particulate exposure

PM_2.5_ was the main particulate exposure characterized in this study, and both occupational and nonoccupational sources were noted. Welding fume, which has a rich content of ultrafine particles (diameter ≤ 0.1 μm) and transition metals, was the main occupational source of PM_2.5_ in our study population (Zimmer 2002). The levels of metal particulates, although expected to be high, were not directly measured because real-time personal monitoring of airborne transition metals was not available. Nonoccupational sources of fine particles included tobacco smoke, food preparation, vehicle exhaust, and ambient air pollution. A DustTrak model 8520 aerosol monitor (TSI Inc., St. Paul, MN) was used to continuously monitor PM_2.5_ within the participant’s breathing zone, and moving averages from ≥ 5 min were generated. Subjects were instructed to wear the monitors while awake and to place the DustTrak on a nightstand while asleep. For participants who slept on the preceding night in the same room as on the sampled night, their previous night PM_2.5_ concentrations were approximated by data from the sampled night. Otherwise, the previous night’s PM_2.5_ data were omitted. Only 4-hr moving averages were used in the statistical analyses, to parallel previous reports (Gold et al. 2000; Magari et al. 2001). We also quantified cross-shift PM_2.5_ exposures using a model 200 personal exposure monitor (PEM; MSP Corporation, Minneapolis, MN) in 2003 and Harvard Cyclones (BGI Inc., Waltham, MA) in 2004 to collect gravimetric air samples. We have documented a good agreement (Spearman’s *r* > 0.90) between real-time readings by DustTrak and gravimetric measures in this occupational setting (Kim et al. 2004).

### Statistical analysis

Because our study subjects were moving freely during concurrently continuous AECG and personal PM_2.5_ exposure monitoring, there were unavoidable time periods when either AECG tracings contained much noise or real-time PM readings were missing. To reduce artifacts of these measurements, we restricted our analyses to AECG segments with > 90% valid beats matched to 5-min epochs with uninterrupted measures of PM_2.5_ in the preceding 4 hr. Also, the length of AECG recording sessions was not uniform across all subjects. As a result, we had an unbalanced data structure with different numbers of repeated measures of 5-min epochs that were not equally spaced in time. Therefore, models with either an exchangeable or a common autoregressive covariance structure, which assume that the time intervals of repeated measures are equal and the same for all individuals, may not be appropriate for our data.

To fully account for the autocorrelation of repeated measures within each subject, we constructed mixed-effect models with a time-dependent covariance structure as an exponential function of temporal distances, such that the correlations of repeated measurements are smaller than observations that were further apart (programming codes available on request). The main effects of 4-hr moving average PM_2.5_ exposure on HRV and HR were first estimated directly from these mixed-effect models, and then the interaction term between PM_2.5_ and obesity was added to evaluate the differential responses between obese and nonobese subjects. In adjusted analyses, we first included age, smoking and drinking habits, calendar year, blood pressure measured at baseline, and obesity. Pulse pressure (i.e., the difference of systolic and diastolic) entered the mixed model, because our empirical data suggested it predicted HRV parameters better than either systolic or diastolic component alone. After accounting for the potential confounding by these time-independent covariates, we then entered each recorded time-varying activity (eating, smoking, coffee drinking, alcohol drinking, exercising, sleeping, and work day) as an indicator variable, and the circadian patterns of HRV and HR were represented by three other indicator variables representing time of day [morning (0700–1100 hr), afternoon (1200–1700 hr), evening (1800–2200 hr), and nighttime (2300–0600 hr)]. All mixed-effects models include a subject-specific random effect to account for any unmeasured between-subject difference in the HRV and HR measures. All these statistical analyses were carried out using SAS 8.0 software package (SAS Institute Inc., Cary, NC), using PROC MIXED procedures and SP(POW) structures with spatial processes replaced by temporal distances. We used the autocorrelation plots of residuals estimated from the final models to evaluate the appropriateness of assumed time-dependent covariance structure. We also carried out sensitivity analyses to assess any potential biases arising from residual confounding by unmeasured time-varying activities, the sensitivity of our results to different model specifications, and the influence of other comorbidities.

## Results

The characteristics of study population are presented in Table 1. No major differences in personal and occupational characteristics were noted between the current study population and the other eligible subjects (*n* = 17). Of the 18 participants of the AECG study, all were male, with 16 white (three Hispanics) and three African Americans. Although 50% (*n* = 9) were classified as obese, no participant had reported or had laboratory evidence of diabetes. None of them were taking any medication at the time of examination. No significant correlation between cross-shift PM_2.5_ concentrations and individual’s BMI was noted (Spearman’s *r* = 0.16, *p* = 0.48). Of all 23 AECG recordings, 12 were collected for 24 hr and 11 were for 48 hr. The SDNN index (mean of standard deviation of all RR intervals for all 5-min segments throughout the entire recordings) was 115.3 ± 7.5 msec and 24-hr average HR was 90.3 ± 1.9 bpm. Higher resting systolic and diastolic blood pressures before the exposure were noted among obese participants than among the nonobese (Table 1). Obese subjects also had slightly higher average HR than nonobese subjects (93.5 ± 2.8 vs. 86.9 ± 2.6 bpm; *p* = 0.07), but their 24-hr SDNN index were similar (117 ± 8 vs. 114 ± 13 msec; *p* = 0.82). Neither the 5-min SDNN (64.5 ± 4.5 msec), rMSSD (52.0 ± 5.2 msec), HF power (801.7 ± 119.1 msec^2^), nor average HR (84.8 ± 2.3 bpm) significantly differed between obese and nonobese subjects. The 4-hr moving average PM_2.5_ concentration was 0.48 ± 0.01 mg/m^3^, ranging from nearly nondetectable to 4.23 mg/m^3^.

### Effects of PM_2.5_ on HR and HRV

Tables 2 and 3 show statistically significant effects, as estimated from mixed-effect models, of PM_2.5_ on autonomic cardiac activities. For each 1-mg/m^3^ increase in 4-hr average PM_2.5_, HR increased by 6.9 bpm [95% confidence interval (CI), 5.0 to 8.7; *p* < 0.0001]; 5-min HRV reduced by 6.6% (95% CI, 2.6 to 10.7) for SDNN (*p* = 0.001), 4.1% (95% CI, –1.8 to 9.9) for rMSSD (*p* = 0.17), and 13.0% (95% CI, 2.0 to 24.0) for HF power (*p* = 0.02), after adjustment for age, smoking status, drinking habit, calendar year, baseline pulse pressure, obesity and time-varying activities (all main effects in model 2 of Tables 2 and 3). There were only few changes to the observed significant associations of PM_2.5_ with average HR and 5-min SDNN after additionally adjusting for the circadian rhythm (morning, afternoon, evening, and night), but the estimated effect on HF power of HRV was diminished (from 13% to 8.8% reduction) and became statistically nonsignificant. Autocorrelation plots did not reveal any remaining temporal correlation among the residuals of constructed mixed effect models with time-dependent covariance structure, indicating a goodness of fit to our data.

### Effect modification by obesity status

We noted that the effects of PM_2.5_ on increases in average HR and reduction of HRV depended on individual’s obesity status, with more aggravated electrophysiologic responses observed in obese subjects. For instance (effect modification in model 3 of Table 2), each 1-mg/m^3^ increase in PM_2.5_ was associated with an 8.7-bpm increase in average HR among obese subjects and a 3.7-bpm increased in nonobese subjects (*p* < 0.001 comparing obese vs. nonobese). Among those with obesity, for each 1-mg/m^3^ increase in PM_2.5_, there were statistically significant associations with a 10.3% reduction in SDNN and 11.1% reduction in HF power of HRV; but the associated 3.4% reduction in rMSSD was statistically nonsignificant. The corresponding responses were 4.0%, 7.2%, and 0.7% for nonobese individuals, and none of these associations were statistically significant (effect modification in all model 3 of Table 3). Only the between-subgroup comparison of the PM-mediated effect on average HR was statistically significant, and a marginally significant difference (*p* = 0.07) was noted for PM_2.5_–SDNN association, probably because of the small sample size. However, there was a very consistent pattern of effect modification by individual’s obesity status across all adjusted models for all time- and frequency-domain measures of HRV. Greater PM_2.5_-mediated HRV reductions (2- to 3-fold differences) in SDNN, rMSSD, and HF power were observed in obese than in nonobese subjects.

### Sensitivity analyses

First, the observed changes to estimated effects of PM_2.5_ on HRV measures (from model 1 estimates to model 2 estimates of Table 3) suggested that PM_2.5_–HRV association could be confounded by time-varying activities. Although we had accounted for several activities in the analyses, we might have missed other important time-varying covariates that correlate both PM_2.5_ exposure and HRV over time. To address this concern with residual confounding, we added to the mixed-effect model (model 2) 23 indicator variables representing the effects of unmeasured activities that presumably affect the SDNN hour by hour. We found that the negative association between PM_2.5_ and SDNN remained statistically significant (–6.6%; 95% CI, –11.3 to –1.9; *p* = 0.006), and the observed SDNN reduction in response to PM_2.5_ was still greater in obese than in nonobese subjects (10.6% vs. 3.9%, *p* = 0.07).

Second, previous air pollution studies on HRV changes have estimated the short-term effects of PM_2.5_ on HRV using different model forms, such as fixed-effect models (Gold et al. 2000; Pope et al. 1999) and generalized estimation equation (GEE) models (Holguin et al. 2003). When we reanalyzed our data by including individual intercepts (fixed-effect models with either 18 or 23 intercepts) or re-estimated PM effects using GEE models assuming a first-order autoregressive correlation among repeated measures, the PM_2.5_–SDNN association remained statistically significant and the difference between obese and nonobese subjects persisted (data not shown).

Third, because obesity and hypertension tend to be clustered together, the observed differential effect of PM_2.5_ on HRV might simply reflect the enhanced susceptibility to PM-mediated HRV reduction among those with hypertension, as reported by others (Holguin et al. 2003; Liao et al. 2004). After we excluded two obese subjects who also had physician-diagnosed hypertension, the observed PM_2.5_ -mediated acute SDNN reduction was still greater among obese subjects (7.1%; 95% CI, 4.9 to 9.4) than the corresponding effect in nonobese subjects (2.0%; 95% CI, –0.1 to 4.0). Finally, few studies on PM–HRV associations had included adjustment for time-varying average HR in the multivariable models (Magari et al. 2001, 2002). Average HR is strongly correlated with ventilation (Mermier et al. 1993; Samet et al. 1993), which is affected by unmeasured physical activities and can influence HRV measures. Therefore, to adjust further for influence of ventilation, we additionally included average HR in the SDNN models. Still, we observed a statistically significant effect of PM_2.5_ on SDNN (7.1% reduction, *p* = 0.003), and the greater response in obese (11.4% reduction in SDNN) versus nonobese subjects (4.3% reduction in SDNN) remained (*p* = 0.06). Sensitivity analyses also yielded consistent findings for effects of PM_2.5_ on average HR and statistically significant effect modification by obesity.

## Discussion

Our study suggests that autonomic cardiac activities in response to PM are differential, depending on individual’s obesity status. In obese subjects (BMI ≥ 30 kg/m^2^), there was an 8.7-bpm increase in HR, whereas the nonobese individuals experienced a 3.7-bpm increase in HR when exposed to the same level (1 mg/m^3^) of PM_2.5_ exposure. Greater PM_2.5_-mediated HRV reductions (2- to 3-fold differences) in SDNN, rMSSD, and HF power were observed in obese subjects, although these differences did not reach statistical significance. These observed alterations in autonomic cardiac activities in response to PM_2.5_ exposure may indicate either activated sympathetic stress response or diminished vagal control. The consistent associations between PM_2.5_ and increased HR and reduced HRV (SDNN and HF), which remained even after adjustment of many time-varying confounders, add to existing literature on the adverse effect of particulate air pollution on autonomic modulation.

The observed differential responses between obese and nonobese individuals support the concept that obesity may impart greater susceptibility to PM-associated acute cardiovascular effects. Recent laboratory data on toxicokinetic features of obese subjects exposed to air pollutants did provide supportive evidence for this concept (Bennett and Zeman 2004). Acute changes in lung mechanics have been suggested to result in the enhanced airway hyperresponsiveness and inflammatory responses to ozone in *ob/ob* obese mice (Rivera-Sanchez et al. 2004; Shore et al. 2003), although the confirmatory data for aggravated PM responses in obese subjects are still lacking. Interestingly, two recent epi-demiologic studies showed that the reduction in pulmonary function measures associated with both short-term and long-term PM exposures were several times higher for obese children/adolescents than those of normal weight (Luttmann-Gibson and Dockery 2004; Occhiuto et al. 2004). In one small panel study on individuals with chronic obstructive pulmonary diseases (*n* = 18) or recent myocar-dial infarction (*n* = 12), it was reported that the positive PM_2.5_–SDNN association increased with decreasing baseline forced expiratory volume in the first second (Wheeler et al. 2006). Whether changes in pulmonary functions also contribute to greater susceptibility to developing PM-mediated acute cardiovascular effects in obese individuals need further investigation.

If confirmed by other studies, the identifi-cation of obesity as a secondary modifier may have mechanistic implications for the cardiovascular effects of air pollution. Airway or parenchymal inflammatory responses to PM have been hypothesized to be the inciting event followed by a cascade of pathophysio-logic changes in autonomic cardiac, systemic inflammation, and hemostatic activities, all of which may ultimately lead to the acute cardiac events associated with PM exposure (Lippmann et al. 2003). More recent data have shown a positive correlation between exhaled nitric oxide, a marker of pulmonary inflammation, and BMI in healthy adults (De Winter-de Groot et al. 2005; Kazaks et al. 2005). Future studies should investigate whether this presumed pulmonary inflamma-tion in obese individuals translates into an enhancement of local inflammatory response to air pollution, thus contributing to the greater PM-mediated HRV reduction in obese individuals.

Previous studies have attempted to identify subpopulations susceptible to acute HRV responses to PM exposure, but the results were varied. Among 21 Boston residents (Gold et al. 2000), smokers were found to have a greater reduction in HRV associated with PM exposures than nonsmokers (*p* = 0.08). A small panel study on 19 subject (9 young and 10 elderly) in Taipei, Taiwan (Chan et al. 2004), revealed a larger decrease in HRV among the elderly with impaired lung functions. Also, there is evidence suggesting that those with hypertension may be more susceptible. In a study among 34 elderly subjects living in Mexico City (Holguin et al. 2003), those with hypertension had a greater reduction in the HF power of HRV in response to PM_2.5_ than those without hypertension. Using data from 497 elderly men in the Normative Aging Study (NAS), investigators showed that the negative associations between PM_2.5_ and HRV were greater for those with hypertension and diabetes mellitus (Park et al. 2005). Because most of these studies were conducted on the elderly population with prevalent comorbidities, it remained unclear whether the observed greater PM–HRV associations were imparted by the comorbid disease processes or attributed to other personal physical characteristics (e.g., obesity) that are closely associated with both hypertension and diabetes mellitus. For instance, in a recent analysis on gene-by-drug-by-environment interaction for PM_2.5_-mediated autonomic effects (Schwartz et al. 2005), descriptive data from 441 subjects in the NAS did suggest that elderly obese people had greater HRV reduction than elderly nonobese subjects. However, it was unclear where such differential response would have persisted, had the analysis accounted for other comorbid conditions (67% hypertensive and 15% diabetic). As indicated in our sensitivity analyses, in this working population of healthy men without overt cardiovascular diseases and diabetes, it is noteworthy that the aggravated response to acute PM_2.5_ in those who were obese remained even after we excluded two participants with both obesity and hypertension.

Exposure characteristics of workplace particulates in our study have implications for future epidemiologic studies on PM-mediated cardiovascular effects. First, PM_2.5_ levels in this occupational setting are much higher than ambient particulate concentrations. Community-based studies need to be conducted to examine whether modification of acute PM effect by obesity status depends on the PM levels. Second, our study findings lend support for ongoing efforts in understanding the cardiotoxicity of metal particulate, especially among the obese subpopulations. Future studies should also examine whether the population susceptibility to PM-mediated cardiovascular effects could be related to differences in the PM compositions, including metal constituents.

We recognize several study limitations. First, measured changes in 5-min HRV reflect only short-term autonomic modulation in response to particulates. Definitive clinical significance of such changes should be elucidated. Second, external validity or generaliz-ability of our results may be limited by the small sample size of 18 subjects. Whether regular exposure to high levels of toxic metal particulates makes subjects more susceptible to acute PM effects than is the general population is unknown, although our ad hoc analyses did not reveal any effect modification by the years of tenure as professional boiler-makers (data not shown). Nevertheless, the internal validity of our findings is supported by the representativeness of our study subjects. Finally, because we did not simultaneously measure other co-pollutants (e.g., ozone, nitrogen dioxide), we failed to directly evaluate the potential confounding by any of these co-pollutants. Also, we were unable to address the possibility that the aggravated HRV reduction in response to PM_2.5_ might be attributable to obese subjects’ enhanced sensitivity to some co-pollutants (e.g., O_3_) which could with PM_2.5_ affect autonomic system activities. Nevertheless, the documented levels of O_3_ and NO_2_ in this occupational cohort were low (Liu et al. 2005), and findings on gaseous co-pollutants in relation to HRV are limited and inconsistent. Personal indoor measures of PM_2.5_ do not covary with personal measures of O_3_, NO_2_ and sulfur dioxide (Sarnat et al. 2000). Thus, the likelihood of such unmeasured confounding is low.

## Conclusions

We found that adverse cardiovascular responses to PM_2.5_ exposures, reflected in reduction of HRV and increases in HR, were aggravated in obese men who did not have overt cardiovascular diseases but were exposed to high levels of metal particulates. These findings support the concept that obesity may impart greater susceptibility to PM-associated acute cardiovascular effects.

## Figures and Tables

**Table 1:** Characteristics of total study population and participants in AECG and personal PM_2.5_ monitoring.

	Total study population				Current study population		
Characteristic	Total population (*n* = 35)	Current study (*n* = 18)	Others (*n* = 17)	*p*-Value^a^	Obese^b^ (*n* = 9)	Nonobese^b^ (*n* = 9)	*p*-Value^a^
Age (years)	38.3 ± 11.7	39.5 ± 9.1	37.1 ± 14.2	0.28	41.8 ± 3.3	37.0 ± 2.4	0.15
Tenure in job (years)	9.7 ± 11.8	8.3 ± 10.0	11.3 ± 13.7	0.84	9.6 ± 3.8	5.6 ± 2.7	0.28
Active smoker (%)	34	39	29	0.73	22	55	0.29
History of hypertension (%)	20	17	23	0.69	22	11	0.53
BMI^b^ (kg/m^2^)	30.2 ± 5.5	30.1 ± 5.3	30.3 ± 5.9	0.90	29.9 ± 1.2	26.7 ± 0.9	< 0.01
Systolic blood pressure (mmHg)	132 ± 13	133 ± 12	131 ± 14	0.77	137 ± 4	126 ± 3	0.007
Diastolic blood pressure (mmHg)	80 ± 9	81 ± 9	80 ± 7	0.68	85 ± 3	75 ± 2	0.003
PEM^c^ PM_2.5_ (mg/m^3^)	1.125 ± 1.072	1.323 ± 1.069	0.913 ± 1.067	0.17	1.639 ± 0.443	1.397 ± 0.313	0.58

Values are mean ± SD or percent.

a *p*-Value for comparing the difference between AECG study subjects and others or between obese and nonobese subjects, given by either rank-sum tests or Fisher’s exact tests.

b Obese: BMI ≥ 30 kg/m^2^; nonobese: BMI < 30 kg/m^2^.

c PEM: personal exposure monitoring for cross-shift PM_2.5_.

**Table 2:** Effects of PM_2.5_ on heart rate in relation to obesity.

	Main effect of PM_2.5_	Modification of PM_2.5_ effect on heart rate		
	bpm change per 1 mg/m^3^ β (95% CI)^a^	BMI < 30 kg/m^2^ (*n* = 9) β (95% CI)^a^	BMI ≥ 30 kg/m^2^ (*n* = 9) β (95% CI)^a^	*p*-Value^b^
Crude analysis	11.0 (10.4–11.5)*	7.6 (6.8–8.4)*	14.9 (14.0–15.7)*	< 0.001
Model 1^c^	11.0 (10.4–11.6)*	7.6 (6.9–8.4)*	14.9 (14.1–15.7)*	< 0.001
Model 2^d^	6.9 (5.0–8.7)*	4.5 (2.1–6.9)*	9.8 (7.2–12.5)*	< 0.001
Model 3^e^	5.9 (4.2–7.7)*	3.7 (1.4–5.9)*	8.7 (6.3–11.2)*	< 0.001

a All regression coefficients and 95% CIs are estimated from mixed-effect models adjusting for autocorrelation.

b *p*-Value comparing obese vs. nonobese subjects.

c Adjusted for age, smoking status, drinking habits, calendar year, blood pressure, and obesity.

d Adjusted for model 1 covariates and time-varying covariates (eating, smoking, coffee drinking, alcohol drinking, exercising, sleeping, and work day).

e Adjusted for model 2 covariates and circadian pattern (morning, afternoon, evening, and night).

* *p* < 0.01 against the null.

**Table 3:** Effects of PM_2.5_ on heart rate variability in relation to obesity.

	Main effect of PM_2.5_	Modification of PM_2.5_ effect on HRV		
HRV measures	% HRV change per 1 mg/m^3^ β (95% CI)^a^	BMI < 30 kg/m^2^ (*n* = 9) β (95% CI)^a^	BMI ≥ 30 kg/m^2^ (*n* = 9) β (95% CI)^a^	*p*-Value^b^
SDNN				
Crude analysis	–4.2 (–7.6 to –0.7)*	–1.1 (–5.9 to 3.7)	–7.6 (–12.6 to –2.5)**	0.07
Model 1^c^	–4.6 (–8.1 to –1.1)**	–1.6 (–6.5 to 3.2)	–7.9 (–12.9 to –2.8)**	0.08
Model 2^d^	–6.6 (–10.7 to –2.6)**	–3.6 (–8.5 to 1.5)	–10.9 (–16.8 to –5.0)**	0.05
Model 3^e^	–6.5 (–11.3 to –1.9)**	–4.0 (–9.5 to 1.5)	–10.3 (–16.7 to –3.9)**	0.07
rMSSD				
Crude analysis	0.4 (–4.7 to 5.6)	1.8 (–5.3 to 9.0)	–1.2 (–8.6 to 6.3)	0.57
Model 1^c^	0.1 (–5.3 to 5.0)	1.1 (–6.0 to 8.3)	–1.5 (–8.9 to 5.9)	0.62
Model 2^d^	–4.1 (–9.9 to 1.8)	–2.3 (–9.7 to 5.0)	–6.5 (–15.0 to 2.0)	0.45
Model 3^e^	–1.7 (–8.4 to 4.9)	–0.7 (–8.6 to 7.3)	–3.4 (–12.6 to 5.9)	0.60
HF				
Crude analysis	–9.2 (–18.8 to 0.4)	–4.6 (–18.0 to 8.8)	–14.3 (–28.2 to –0.4)*	0.32
Model 1^c^	–10.6 (–20.3 to –0.9)*	–6.6 (–20.0 to 6.8)	–14.9 (–28.8 to –1.1)*	0.39
Model 2^d^	–13.0 (–24.0 to –2.0)*	–10.4 (–24.2 to 3.4)	–16.5 (–32.5 to –0.6)*	0.55
Model 3^e^	–8.8 (–21.3 to 3.8)	–7.2 (–22.2 to 7.8)	–11.1 (–28.4 to 6.2)	0.70

a All regression coefficients and 95% CIs are estimated from mixed-effect models adjusting for autocorrelation.

b *p*-Value comparing obese vs. nonobese subjects.

c Adjusted for age, smoking status, drinking habits, calendar year, blood pressure, and obesity.

d Adjusted for model 1 covariates and time-varying covariates (eating, smoking, coffee drinking, alcohol drinking, exercising, sleeping, and workday).

e Adjusted for model 2 covariates and circadian pattern (morning, afternoon, evening, and night).

* *p* < 0.05;

** *p* < 0.01 against the null.
